# A bioreactor-based platform for investigating the early response of human periodontal ligament stem cells to intermittent mechanical stretching

**DOI:** 10.3389/fbioe.2025.1634143

**Published:** 2025-09-03

**Authors:** Giovanni Putame, Beatrice Masante, Marta Tosini, Andrea T. Lugas, Ilaria Roato, Mara Terzini, Alberto L. Audenino, Federico Mussano, Diana Massai

**Affiliations:** ^1^ Department of Mechanical and Aerospace Engineering and Polito^BIO^Med Lab, Politecnico di Torino, Turin, Italy; ^2^ Interuniversity Center for the Promotion of the 3Rs Principles in Teaching and Research, Turin, Italy; ^3^ Bone and Dental Bioengineering Lab, Department of Surgical Sciences, CIR-Dental School, University of Turin, Turin, Italy

**Keywords:** deformable substrate, bioreactor-based investigation platform, intermittent stretching, mechanical characterization, periodontal ligament stem cells, tissue engineering

## Abstract

During development and daily activities, biological tissues are frequently exposed to mechanical stimuli, which are crucial for tissue maintenance and regeneration. The periodontal ligament (PDL), which connects the tooth root to the alveolar bone of the jaw, is among the tissues most exposed to mechanical loading and has recently received increasing attention due to the rising prevalence of periodontitis, a chronic inflammatory disease that leads to the progressive destruction of tooth-supporting structures. Understanding the mechanobiology of PDL could be essential for guiding effective regenerative strategies. To address this, a bioreactor-based platform for applying controlled stretch stimulation to adherent cells was developed, and the early biological response of human primary PDL stem cells (hPDLSCs) to different intermittent stretching protocols was investigated. Furthermore, to correlate the mechanical stimulus applied to the cells with their biological response, a detailed characterization of the substrate deformation was performed. The platform integrates an existing stretch bioreactor, updated to enable automated alternation of constant and dynamic stretching conditions without user intervention, with a custom-designed polydimethylsiloxane (PDMS) deformable substrate, whose geometry was optimized for ensuring the most uniform strain distribution. The mechanical behavior of the substrate was accurately characterized via finite element analyses and experimental tensile tests combined with digital image correlation analyses. This revealed slight discrepancies between the imposed and actual strain experienced by the substrate and assumed to be provided to the adherent cells. Preliminary biological experiments showed distinct responses in hPDLSCs and adipose-tissue derived stem cells (ASCs) exposed to intermittent stretching: hPDLSCs exhibited upregulation of osteogenic gene expression, while ASCs showed no significant changes under identical conditions. Furthermore, hPDLSCs were exposed to three different intermittent stretching protocols. Increasing the total daily cyclic stretch exposure enhanced the hPDLSCs early response, including alignment along the stretch direction and upregulation of both osteogenic and PDL-related gene expression. Overall, this study confirmed the suitability of the proposed platform for investigating the effects of controlled stretching on mechanosensitive cells such as hPDLSCs and provided valuable insights into their early response to intermittent stretching protocols.

## 1 Introduction

*In vivo*, cells are exposed to a dynamic biomechanical environment, characterized by various forces (e.g., stretch, compression, shear stress) and physical cues depending on the biological tissue (Subramanian et al., 2017). Over the past two decades, mechanobiology studies have demonstrated that mechanical stimuli play a crucial role in regulating cell proliferation and differentiation, influencing tissue remodeling, homeostasis, and even the pathogenesis of several diseases (Mammoto et al., 2012; Martino et al., 2018; Stewart et al., 2020). Furthermore, in tissues characterized by oriented fibers and organized structure, such as bone, muscle, and ligament, mechanical stimuli guide cell alignment.

One of the tissues most exposed to mechanical stimulation and recently widely investigated (Zhao et al., 2022) is the periodontal ligament (PDL), which connects the tooth root to the alveolar bone of the jaw. During the normal oral functions, such as mastication or speaking, the PDL undergoes a continuous and variable combination of compression, stretch, and shear stress stimuli (Roato et al., 2022). The mechano-biological response of the PDL to these loading conditions determines the orientation of the PDL collagen fiber bundles in a horizontal, oblique, or vertical direction, depending on their location along the tooth root (Gauthier et al., 2021). Interestingly, the turnover of mature collagen fibers in the PDL is reported to be extremely fast, i.e., from 1 to 6 days depending on the considered region of PDL (Sodek, 1977). Moreover, a controlled mechanical loading of the PDL and the surrounding alveolar bone, such as during orthodontic treatments, promotes coordinated bone formation on the traction side and bone resorption on the compression side, leading to the reorganization of the jaw bone tissue (Sun et al., 2021). In the case of periodontitis, a progressive inflammatory pathology that represents a widespread and growing health concern (Kwon et al., 2021), the PDL undergoes significant degradation, impairing its ability to transmit mechanical loads effectively. This degenerative process leads to alveolar bone resorption and, ultimately, tooth mobility or loss (Papapanou et al., 2018). Understanding the mechanobiological response of the PDL under both physiological and pathological mechanical conditions would be crucial for developing effective therapeutic strategies aimed at preserving or regenerating periodontal health and function. However, traditional monolayer cell cultures, performed on rigid substrates under static conditions, are inadequate for investigating *in vitro* the biological response of PDL cells and tissue to the dynamic mechanical stimuli characteristic of their native environment (Chen et al., 2015; Wang et al., 2022).

In the past, a plethora of bioreactors combined with deformable substrates have been developed and employed to transmit native-like mechanical stimuli to adherent cells (Bighi et al., 2025; Jiangtao et al., 2023; Leung et al., 1977; Salim et al., 2022; Meng et al., 2022; Liu et al., 2017; Papadopoulou et al., 2016). This has opened new avenues for mechanotransduction studies, aimed at elucidating cellular responses to specific mechanical cues and uncovering the complex signaling pathways involved (Leung et al., 1977; Liu et al., 2017; Kamal et al., 2024; Basak et al., 2024; Martin et al., 2004; Massai et al., 2013; Ravichandran et al., 2018; Gabetti et al., 2022; Yeatts et al., 2013; Jiang et al., 2021; Shi et al., 2022). Nevertheless, most stretch devices reported in the literature offer limited automation and low tunability of stimulation parameters. Furthermore, a thorough characterization of the actual mechanical environment to which cells are exposed is often lacking, which limit the ability to accurately correlate mechanical stimuli with the resulting biological responses.

For the production of stretchable substrates, silane-based silicones have been widely used, with polydimethylsiloxane (PDMS) being one of the most adopted elastomeric material due to its high biocompatibility and versatility (Friedrich et al., 2017; Esmaeili et al., 2024; Hassler et al., 2011). PDMS can be easily casted in different tailored shapes and, in combination with bioreactors, used as a deformable substrate on which the cells can adhere and be subjected to controlled uniaxial, biaxial, or circumferential stretch (Friedrich et al., 2017). Nevertheless, PDMS substrates present some limitations, including intrinsic surface hydrophobicity, which impairs cell adhesion and requires additional surface functionalization. Moreover, their mechanical properties are sensitive to the manufacturing process, material composition, sterilization process and geometrical variations, which may lead to unexpected or non-uniform strain distribution during mechanical stimulation, ideally requiring prior mechanical characterization (Mata et al., 2005).

In mechanobiology studies, it is of the utmost importance to tune the parameters of the stimulation (e.g., amplitude, frequency, and waveforms) in order to guarantee high accuracy, reproducibility, and replicability of experiments. In 2013, Saminathan et al. found that cyclic equibiaxial stretching (12%, 0.2 Hz) induced PDL extracellular matrix (ECM) remodeling by upregulating the matrix metalloproteinase-1 (MMP1) expression (Saminathan et al., 2013). Chen et al. confirmed increased ECM gene expression under similar conditions (Chen et al., 2015). Additionally, cyclic stretching has been shown to induce osteogenic differentiation in human-derived periodontal ligament stem cells (hPDLSCs), with variations in duration and frequency influencing differentiation outcomes (Meng et al., 2022; Shen et al., 2014; Wei et al., 2015; Wang et al., 2019; Xi et al., 2021). Xi et al. reported that stretching (10%, 0.5 Hz) elevated reactive oxygen species (ROS) levels, potentially contributing to osteogenesis (Xi et al., 2021), while Liu et al. (2017) found that hPDLSCs from periodontitis patients exhibited greater sensitivity to static strain (6%–14%) compared to those from healthy donors (Liu et al., 2017). Notably, de Jong et al. (Jong et al., 2017) in their study employed an intermittent cyclic stretching protocol designed to mimic both the dynamic external periodontal loading from mastication and the continuous intrinsic strain exerted on the PDL fibers by PDL fibroblasts. However, these studies applied mechanical stimuli with differing magnitudes, frequencies, and durations, making direct comparisons challenging. Furthermore, most did not characterize the actual strain experienced by the cells, limiting the ability to accurately correlate specific mechanical inputs with defined cellular responses. Indeed, to establish precise mechanobiological relationships, it is crucial to characterize and control the strain field generated on the substrate as well as the deformation experienced by adherent cells (Constantinou and Bastounis, 2023).

For this purpose, experimental and numerical engineering approaches have been adopted (Ribeiro et al., 2019). For instance, Quinlan et al. (2011) characterized the strain distribution within a flexible two-dimensional (2D) culture system to ensure accurate mechanical stimulation of cells. Using digital image analysis and high-density mapping (HDM), the researchers confirmed that the applied strain was uniformly distributed within the central region of the substrate (Quinlan et al., 2011). Subramanian and colleagues demonstrated the uniform uniaxial strain on the exploited constructs by means of finite element (FE) analysis (Subramanian et al., 2017). However, as reported by Saminathan et al., when a substrate deforms, cells experience a mix of tensile, compressive, and shear strains, with the deformation varying depending on their position (Saminathan et al., 2013). Moreover, it was demonstrated that in some commercial systems, the actual deformation of the substrate is about half of what is expected, which can impact the interpretation of cellular responses to mechanical stimuli (Geest et al., 2004; Wall et al., 2007). The combination of computational and experimental methods could enable a comprehensive characterization of the materials and stimulation conditions, providing a deeper understanding of the material behavior and improving the ability to simulate physiological conditions *in vitro* (Ribeiro et al., 2019). Thus, the complete characterization of strain distribution on deformable substrates or three-dimensional (3D) constructs could significantly improve the understanding of the relationship between the actual applied strain and the cellular response (Geest et al., 2004).

Inspired by this framework, we developed a bioreactor-based platform for culturing and studying *in vitro* adherent cells under tunable intermittent stretch conditions, and we investigated the early biological response of hPDLSCs to different intermittent stretching protocols. Specifically, a PDMS deformable substrate, to be used in combination with a uniaxial stretch bioreactor (Massai et al., 2020; Putame et al., 2020), was designed, optimized, and mechanically characterized through a combined computational-experimental approach, integrating FE modeling with uniaxial tensile tests and digital image correlation (DIC) analyses. This integrated approach allowed to accurately quantify the actual strain fields experienced by the substrate, providing an informed basis into the mechanical environment to which the adherent cells were exposed. Moreover, the control software of the bioreactor was updated for the automated delivery of intermittent constant and dynamic stretching conditions, without requiring user intervention. Preliminary biological experiments with hPDLSCs and adipose-derived stem cells (ASCs) revealed cell-type-specific responses to intermittent stretching, contributing to a better understanding of their potential applications in regenerative medicine. Lastly, hPDLSCs were exposed to three distinct intermittent stretching protocols, differing in cyclic stretching duration and stimulation interval, and their early biological response was assessed in terms of cell orientation and osteogenic and PDL-related gene expression.

## 2 Materials and methods

### 2.1 Bioreactor-based platform

**FIGURE 1:**
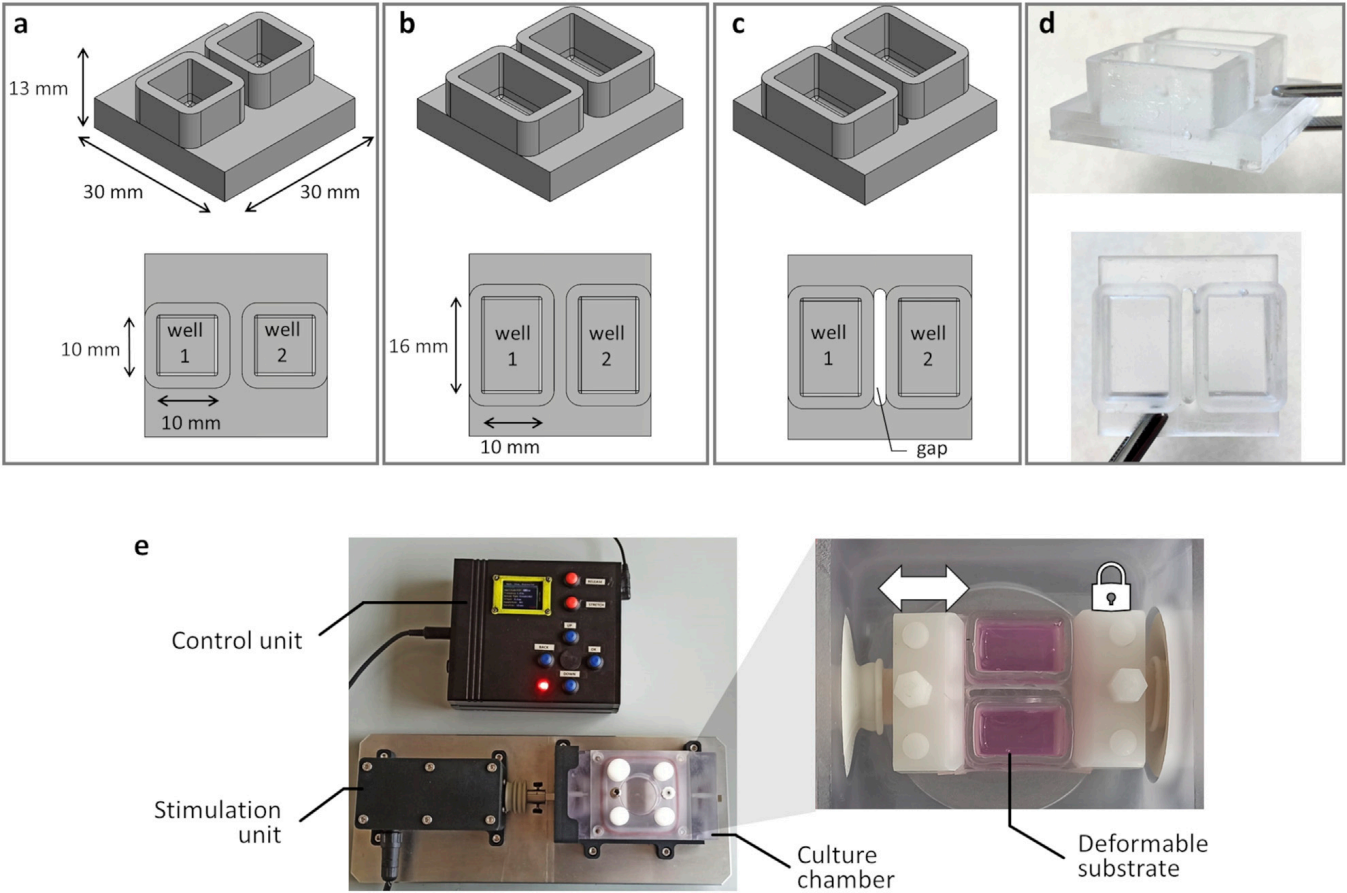
Deformable substrate versions and investigation platform. 3D models of the substrate versions and top view (on bottom) showing the substrate geometrical features and dimensions: **(a)** S1 version; **(b)** S2 version; **(c)** S3 version. **(d)** S3 version manufactured in PDMS. **(e)** Investigation platform composed of the automated stretch bioreactor, with the control unit, the stimulation unit, and the culture chamber, housing the S3 mounted in the clamping system.

The proposed investigation platform was developed for exposing adherent cells to tunable, controlled stretch and consists of a custom-designed deformable substrate to be used in combination with an automated stretch bioreactor, previously developed (Massai et al., 2020; Putame et al., 2020). For the deformable substrate, three versions (Figure 1) were designed (Solidworks 2021, Dassault Systemes, France) supported by FE analysis for assessing the influence of the substrate geometrical features on its deformation under stretch and for selecting the optimal configuration (see $2.2.1). In detail, the maximum substrate dimensions (30 mm × 30 mm × 13 mm) were determined based on the constraints imposed by the bioreactor culture chamber and clamping system. Furthermore, for parallelizing the test while minimizing the culture medium volume, each substrate was designed with two parallel wells. The first version of the deformable substrate (S1) was designed with squared wells (10 mm × 10 mm × 9 mm; surface area = 100 mm^2^; priming volume = 0.90 mL, Figure 1a). To expand the available culture surface, the second version (S2) presented rectangular wells (10 mm × 16 mm × 9 mm; surface area = 160 mm^2^; priming volume = 1.44 mL, Figure 1b). To ensure uniform and symmetric strain within each well, the third version (S3) retained the S2 rectangular well design but a gap was introduced along the stretch direction for separating the wells (Figure 1c). Based on the FE outcomes (see $3.1), S3 was selected as the optimal layout and was then manufactured in-house (Figure 1d). For substrate manufacturing, a dedicated mold was designed and 3D-printed by stereolithography (Grey Resin, FormLabs, USA). The PDMS silicone (Sylgard 184, Dow Chemical, USA) was then selected for its cytocompatibility, autoclavability, and deformability, prepared at a 10:1 ratio, poured into the mold, and cured at 60 °C for 5 h.

As regards the bioreactor (Massai et al., 2020; Putame et al., 2020), briefly it consists of three main units (Figure 1e): (i) the culture chamber, where the deformable substrate is clamped; (ii) the stimulation unit, for providing tunable uniaxial cyclic stretch; (iii) the control unit, for setting and automatically controlling the stimulation parameters (displacement = 0.1–3.0 mm; frequency = 1–3 Hz; sinusoidal or triangular waveform). Within the culture chamber, two opposite clamps secure the substrate in place, and the clamp connected to the stimulation unit actuator enables substrate stretching when the actuator is activated (Figure 1e). For this study, the control unit software was updated for delivering intermittent stretch stimulations, enabling the user to set stimulation intervals (ranging from 1 to 300 s every 1–24 h) for alternating constant and dynamic stretching without user intervention.

### 2.2 Mechanical characterization of the substrate

To support the development and the selection of the deformable substrate, both computational and experimental analyses were performed and the results were compared.

#### 2.2.1 Computational analysis

During the substrate design process, a preliminary FE analysis was performed to characterize its deformation under uniaxial stretch and to select the geometry that ensures maximum strain uniformity. In detail, static simulations were carried out by using the software COMSOL Multiphysics 5.3 (Comsol Inc., USA). For each substrate version, the geometry was meshed using linear tetrahedral elements (∼8·10^4^) and the mechanical properties of the PDMS were imposed (linear-elastic material, Young’s modulus = 1.5 MPa, Poisson’s ratio = 0.49 (Johnston et al., 2014)). For this preliminary investigation, the clamped zones of the substrate (5 mm in length for each side) were neglected and the boundary conditions were applied to the remaining ends: a linear displacement of 3 mm along the stretching direction was imposed to the moving end while the opposite end was constrained. The three substrate versions were compared in terms of strain distribution at the well bottom, particularly focusing on the strain components parallel and perpendicular to the stretch direction. The out-of-plane displacement of the well bottom was also computed.

**FIGURE 2:**
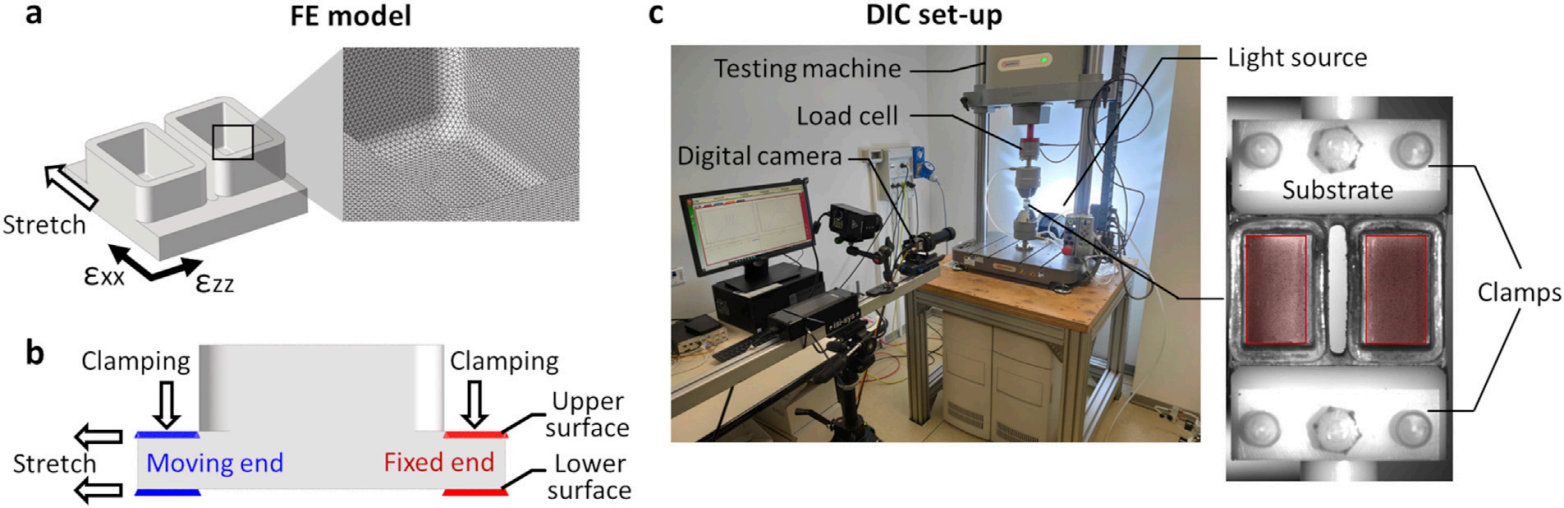
Mechanical characterization of S3 samples by computational and experimental methods. **(a)** Schematic drawing of the S3 sample geometry with strain reference directions and mesh (detail box). **(b)** Schematic drawing of the lateral view of the S3 sample, showing the boundary conditions applied during the clamping phase and the stretching phase with constrained (red) and moving (blue) nodes. **(c)** Experimental setup for mechanical characterization of the S3 samples. On the left, universal testing machine and digital image correlation (DIC) system. On the right, picture of the S3 sample acquired for DIC analysis showing the applied speckle. Red areas indicate the regions of interest (ROI) considered for strain computation.

Once the S3 version was identified as the geometry ensuring the maximum strain uniformity, a refined FE analysis was conducted for fully characterizing the behavior of the deformable substrate under uniaxial stretching. The S3 design was meshed (HyperMesh 2017, Altair Engineering, USA) with linear tetrahedral elements (Tetra 4, 7.7·10^5^ elements, element size = 0.1 mm, Figure 2a) and a static FE simulation was carried out (Abaqus 2017, Dassault Systèmes, France) setting the PDMS properties previously adopted. The simulation involved two sequential phases: initial clamping of the substrate followed by stretching. In detail, for the clamping phase, a linear displacement of 0.6 mm perpendicular to the substrate surface was imposed on the nodes at both ends of the upper surface, while the nodes on the lower surface were constrained (Figure 2b). Successively, for the stretching phase, the selected nodes on the end connected to the actuator were translated of 3 mm along the stretch direction, while the nodes on the opposite end remained constrained. The strain distributions on the upper surface of the substrate were computed, together with their mean values, and presented using color maps. Specifically, the strain components parallel (ε_xx_) and perpendicular (ε_zz_) to the stretching direction were observed separately at the maximum stretch. Moreover, relative frequencies of the strain values were presented as histograms.

#### 2.2.2 Experimental analysis

Before performing the experimental mechanical characterization tests on S3 version, the samples underwent three autoclave cycles to exclude potential effects of the sterilization process on the substrate’s mechanical response. For the tests, a universal testing machine (Model E3000, Instron, USA) was used with the bioreactor clamping system mounted on it using pneumatic grips (Figure 2c). For each S3 sample, three stretching tests in displacement control were performed (S3, n = 3). Each test included a manual clamping phase, followed by a pre-elongation of 1.6 mm (8% strain) at a rate of 0.5 mm/s, and the application of 90 stretch cycles with a maximum displacement of 3 mm (15% total strain) at a frequency of 1 Hz. The applied traction force was recorded over time at a sampling rate of 25 Hz using a load cell (Dynacell 2527 Series, Instron, ±5 kN full scale range, 0.5% accuracy at 40 N). The maximum traction force was determined as the mean of the measured force peaks using a MATLAB custom script (R2022a, MathWorks, USA).

For measuring the strain distribution on the surface of the S3 samples while subjected to deformation, a full-field non-invasive optical DIC analysis was performed (Figure 2c). In detail, a VIC-2D system was used (isi-sys GmbH, Germany) including one camera (9 MegaPixels, 4096 × 2168 pixels, 8-bit, black-and-white), and a random black speckle pattern was created on the surface of the S3 samples using a spray can. The camera was positioned at approximately 690 mm from the sample, obtaining a 50 mm × 30 mm field of view and a pixel size of 0.012 mm. Images acquisition (25 frames/s) was synchronized with the applied stretching using an external trigger signal. To avoid artefacts due to light reflection and to enhance the image contrast, different lighting conditions were tested by adopting direct and indirect lighting (LED 650, isi-sys GmbH, Germany). The best lighting condition was obtained illuminating the substrate using a white LED indirect backlight. The DIC system was calibrated using a calibration target, and the strain distribution was then computed using the VIC-2D software (isi-sys GmbH, Germany), with the bottom of the wells defined as the region of interest (ROI). The parameters adopted for the DIC analysis are listed in Supplementary Table S1. The strain distribution on the substrate at the maximum stretch was depicted separately for ε_xx_ and ε_zz_ by using color maps. Additionally, relative frequencies of the strain values, averaged over the stretch cycles, were computed and presented for comparison with the FE results.

### 2.3 Biological experiments

Following the manufacturing and the mechanical characterization of the S3 substrate, biological tests were conducted to assess (*i*) the cell-type-specific response of hPDLSCs and ASCs to intermittent stretching, and (*ii*) the early biological response of hPDLSCs to three distinct intermittent stretching protocols, differing in cyclic stretching duration and stimulation interval.

#### 2.3.1 Cell sources

Two cell types were used, namely, primary hPDLSCs harvested from four healthy donors in accordance with ethical principles (protocol No. 0107683, 3 October 2022, approved by Comitato Etico Interaziendale A.O.U. Città della Salute e della Scienza di Torino–A.O. Ordine Mauriziano–A.S.L. “Città di Torino”) and ASCs from cell line (ASC52 – telo hTERT, ATCC). In detail, hPDLSCs were obtained from explanted wisdom teeth by scratching the tooth root with a scalpel, the small fragments were collected and washed with physiological saline buffer (PBS, Life Technologies, USA), then digested for 10 min with a solution of 1 mg/mL dispase II (PluriSTEM™, Merck, Germany) and 3 mg/mL collagenase I (Sigma-Aldrich, USA). The retrieved cells were seeded in a 75 cm^2^ flask and cultured for at least 5 days in α-MEM (Life Technologies, USA) added with 5% of human platelet lysate (hPL, IsoCell Growth, Euroclone, Italy), 1% penicillin (100 U/mL)-streptomycin (100 μg/mL) (Life Technologies, USA), and 1% fungizone (Life Technologies, USA). The hPDLSCs were expanded and cryopreserved until use. The ASCs were selected for two main reasons: (1) they are widely used in a variety of tissue engineering studies, due to their multilineage differentiation potential and ease of isolation (Jong et al., 2017; Li and Guo, 2018); and (2) as a non-dental cell type, they served as a benchmark for comparing the mechanobiological behavior of two stem cell populations with different tissue origins.

#### 2.3.2 Stretch stimulation protocols

**FIGURE 3:**
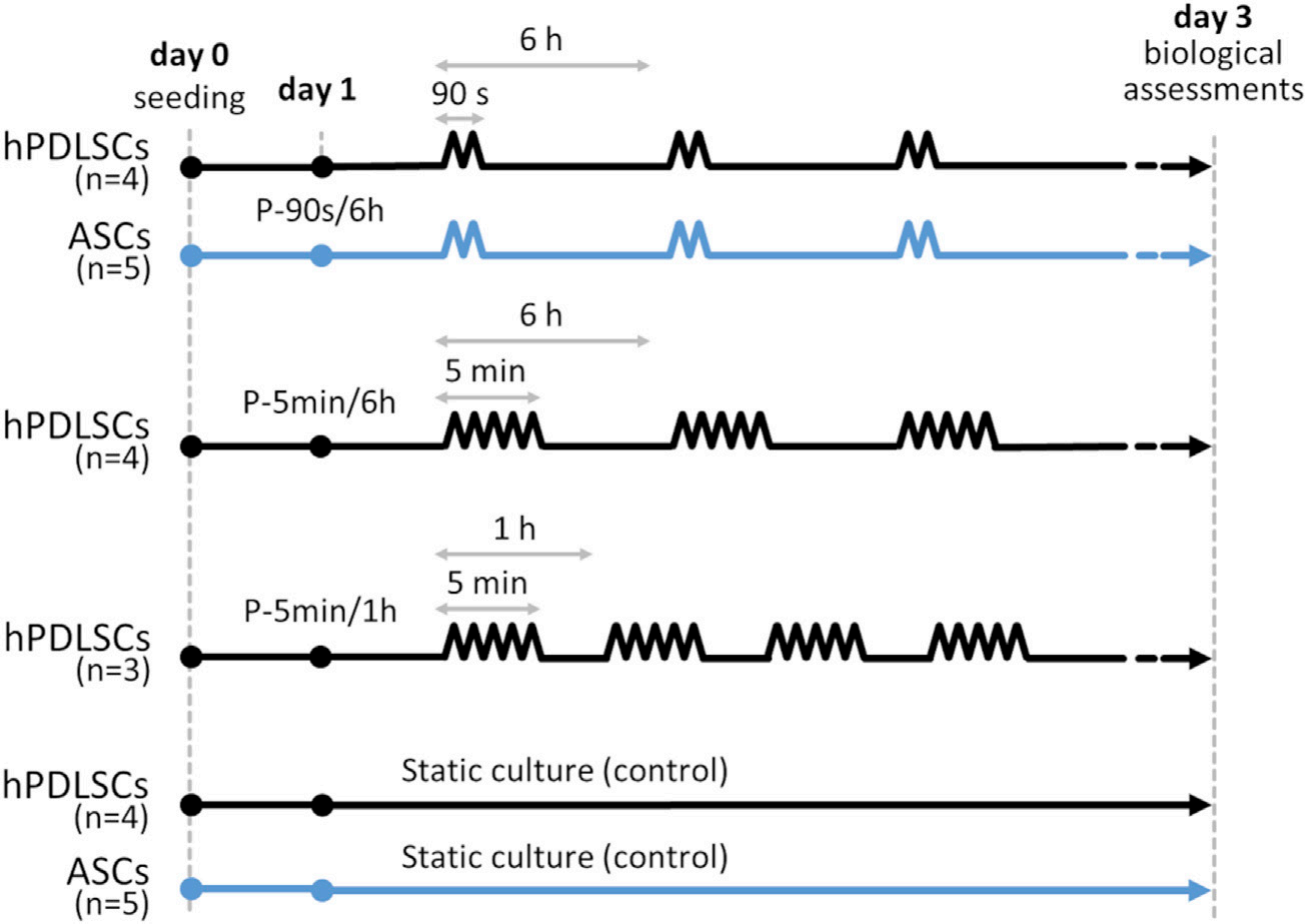
Timeline of the culture protocols applied on hPDLSCs (black lines) and ASCs (light blue lines). P-90s/6 h protocol: 8% of constant strain plus 7% cyclic strain (1 Hz, triangular waveform) for 90 s every 6 h for 3 days; P-5 min/6 h protocol: 8% of constant strain plus 7% cyclic strain (1 Hz, triangular waveform) for 5 min every 6 h for 3 days; P-5 min/1 h protocol: 8% of constant strain plus 7% cyclic strain (1 Hz, triangular waveform) for 5 min every 1 h for 3 days; controls: static conditions for 3 days.

To promote cell adhesion to the bottom of the substrate well, this latter was coated with 50 µg/mL type I collagen (Cultrex™, R&D System, USA). Briefly, the collagen was diluted in acetic acid (Carlo Erba, Italy) 0.02 M and poured into the substrate wells, then it was incubated at 37 °C for 4 h and rinsed three times with PBS. Thereafter, both for hPDLSCs and ASCs, 1 × 10^5^ cells (62.5 × 10^3^ cells/cm^2^) were seeded in each well and 1 mL of α-MEM was added. After 24 h in incubator (37 °C, 5% CO_2_) to favor cell adhesion, the stretching stimulation was applied. Inspired by the work of de Jong (Jong et al., 2017), an intermittent stretching protocol, designed to mimic the continuous intrinsic strain of the periodontium and the dynamic external loading associated with mastication, was applied both to hPDLSCs (n = 4) and ASCs (n = 5). The protocol, identified as P-90s/6 h, consisted of 8% constant strain (representing the intrinsic PDL strain) combined with 7% cyclic strain (1 Hz, triangular waveform) for 90 s every 6 h (mimicking the dynamic stretching associated with mastication) for 3 days (Figure 3; Masante et al., 2023). As control, hPDLSCs (n = 4) and ASCs (n = 5) were seeded into the substrate wells and cultured in static condition within the incubator for 3 days. Subsequently, to investigate the role of stretching parameters on the early response of hPDLSCs, particularly the influence of the cyclic stretching associated with mastication, two additional intermittent stretching protocols were tested. For these, the same constant strain and cyclic strain amplitudes of P-90s/6 h were maintained, but the duration and interval of cyclic stretching stimulation were modified. This allowed the assessment of how extended and more frequent dynamic stretching affects hPDLSC behavior (Figure 3). The protocols were as follows: P-5 min/6 h (n = 4), 8% constant strain combined with 7% cyclic strain (1 Hz, triangular waveform) for 5 min every 6 h for 3 days, designed to deliver mastication-associated cyclic stretching over a more physiologically relevant timeframe compared to P-90s/6 h; P-5 min/1 h (n = 3), 8% constant strain combined with 7% cyclic strain (1 Hz, triangular waveform) for 5 min every 1 h for 3 days, intended to explore the effects of more frequent mastication-like cyclic stretching.

#### 2.3.3 Biological assessments

##### 2.3.3.1 Comparison among hPDLSCs and ASCs cultured under P-90s/6 h stretching protocol

A preliminary biological assessment concerned the comparison of the effects of the intermittent stretching stimulation P-90s/6 h on hPDLSCs and ASCs. In detail, after culture, both hPDLSCs and ASCs cultured under dynamic and static conditions were treated with TRIzol reagent (ThermoFisher Scientific, USA) for RNA extraction. Specifically, 1 µg of RNA was converted up to single-stranded cDNA using the High-Capacity cDNA Reverse Transcription Kit (Applied Biosystems, USA). Quantitative real-time PCR was performed by the CFX96 system (Bio-Rad, USA) with the Luna Universal qPCR Master Mix (New England BioLabs Inc., USA). The amplification protocol foresees 40 cycles with a melting temperature of 60 °C, and β-actin was chosen as housekeeping gene for normalizing gene expressions data. The 2^−ΔΔCT^ method was adopted for quantitative analysis. For the comparison among hPDLSCs and ASCs, the expression of the osteogenic markers alkaline phosphatase (ALP), collagen type I (COLI), osteocalcin (OCN), and runt-related transcription factor 2 (RUNX2) was analyzed.

##### 2.3.3.2 Assessment of the early biological response of hPDLSCs cultured under three different intermittent stretching protocols

Subsequently, the early response of hPDLSCs to three distinct intermittent stretching protocols, varying in cyclic stretching duration and stimulation interval, was investigated. To assess cell orientation, brightfield images of the central region of the wells with adhered hPDLSCs were collected immediately after the dynamic and static cultures with the JuLiTM Br Smart bright-cell movie analyzer (NanoEntek, Korea). To ensure reproducibility during image acquisition, the substrates were consistently positioned by marking the longitudinal axis of the well (aligned with the stretching direction) and placing the displaced edge, connected to the actuator, at the top. A preliminary assessment of cell orientation (expressed as angles, with 0° corresponding to the stretch direction) was performed on one representative image per condition, using the OrientationJ plugin in Fiji (ImageJ, version 2.0). Subsequently, a statistical analysis of cell orientation distribution relative to the stretch direction was conducted for static control (n = 4), P-5 min/6 h (n = 4), and P-5 min/1 h (n = 3) culture protocols, based on images acquired from independent samples. The cell orientation angles were exported from Fiji and, for each value, the deviation from the stretching direction was calculated (using absolute values) and then averaged across replicates. To compare the cell orientation distributions, the results are presented as violin plots, with the angle range limited between 0° (corresponding to the stretch direction) and 90°, displaying both mean and median values. Moreover, hPDLCs were treated for RNA extraction following the procedure previously described and the expression of both osteogenic markers (ALP, COL1, OCN, RUNX2, and Osterix (OSX)) and mechanoresponsive markers associated with the PDL (collagen type XII (COL XII), Decorin and Periostin) (Hosiriluck et al., 2022; Izu and Birk, 2023) was analyzed.

#### 2.3.4 Statistical analysis

For the PDMS substrate characterization, the comparison between the computational and experimental strain values was statistically analyzed adopting the two-tailed one-sample t-test using MATLAB (R2022a, MathWorks, USA). For the gene expression, the statistical analysis was performed using GraphPad Prism 8.0 (GraphPad Software Inc., USA). Data were analyzed by t-test. Results are presented as mean ± standard deviation. For cell orientation assessment, the non-parametric Kruskal-Wallis test followed by Dunn-Šidák *post hoc* test was performed, using MATLAB (R2022a, MathWorks, USA). For each analysis, results were considered statistically significant with *p*-value < 0.05.

## 3 Results

Concerning the bioreactor-based platform, the design of the PDMS deformable substrates, supported by computational modeling, and the consequent manufacturing process demonstrated to be effective. In particular, the S3 version was used in combination with the stretch bioreactor, resulting in a platform able to deliver intermittent stretch stimulation to adherent cell cultures. To characterize the stimulation provided by the platform, the mechanical behavior of the substrate during stretching was investigated in terms of deformation using both computational and experimental approaches. Moreover, exploratory biological tests were carried out on hPDLSCs and ASCs to test the performances of the platform, to assess the effects of intermittent stretching stimulations on different stem cells, and to investigate the early biological response of hPDLSCs to distinct intermittent stretching protocols. The following sections illustrate the mechanical characterization and the biological results.

### 3.1 Mechanical characterization of the substrate

**FIGURE 4:**
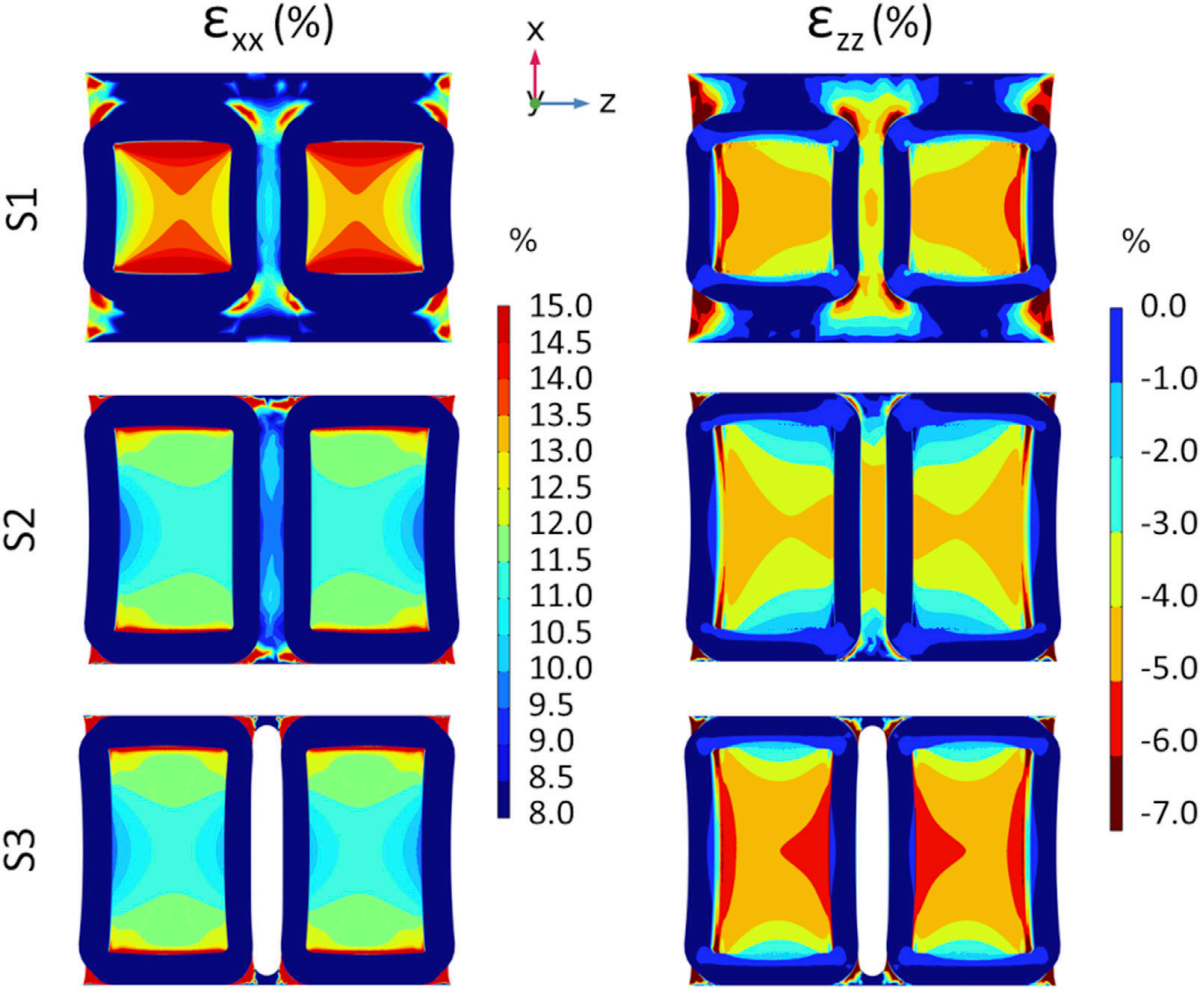
Results from preliminary FE analyses showing the color maps of strain distribution at the S1 (top row), S2 (middle row), and S3 (bottom row) well bottoms at the maximum stretch amplitude of 3 mm. Strain distribution along the stretching direction (ε_xx_, on the left) and in the perpendicular direction (ε_zz_, on the right).

The preliminary FE analyses performed on the three versions of the substrate, aimed at evaluating their deformation under uniaxial stretch, allowed identifying the optimal geometry in terms of strain uniformity and symmetry. Figure 4 shows the strain distributions, along (ε_xx_) and perpendicular (ε_zz_) the stretching direction, at the bottom of the three different substrate versions (S1, S2, and S3). The S1 version exhibits the highest strain values along the stretch direction (ε_xx_), with the well bottom experiencing strains of up to 13%–14%, though only in localized regions. Differently, the S2 and S3 versions exhibit strain values ranging between 11% and 12%, characterized by a more uniform distribution. In the case of S3, the distribution also appears symmetric, due to the presence of the gap between the wells. Looking at the strain perpendicular to the stretch direction (ε_zz_), all versions showed strain values ranging between −6% and −3%. This phenomenon derives from the PDMS Poisson’s ratio (ν = 0.49), which indicates that a tensile stress in one direction results in compression in the perpendicular direction. Concerning the out-of-plane deflection (ε_yy_), all versions showed negligible strain values, with a maximum displacement of about 0.1 mm (results not shown). Given the more uniform and symmetric strain distribution at the bottom of S3 wells compared to S1 and S2, along with the importance of sufficient surface area for cell adhesion to ensure reliable biological analyses, S3 was selected as the optimal substrate to be manufactured.

**FIGURE 5:**
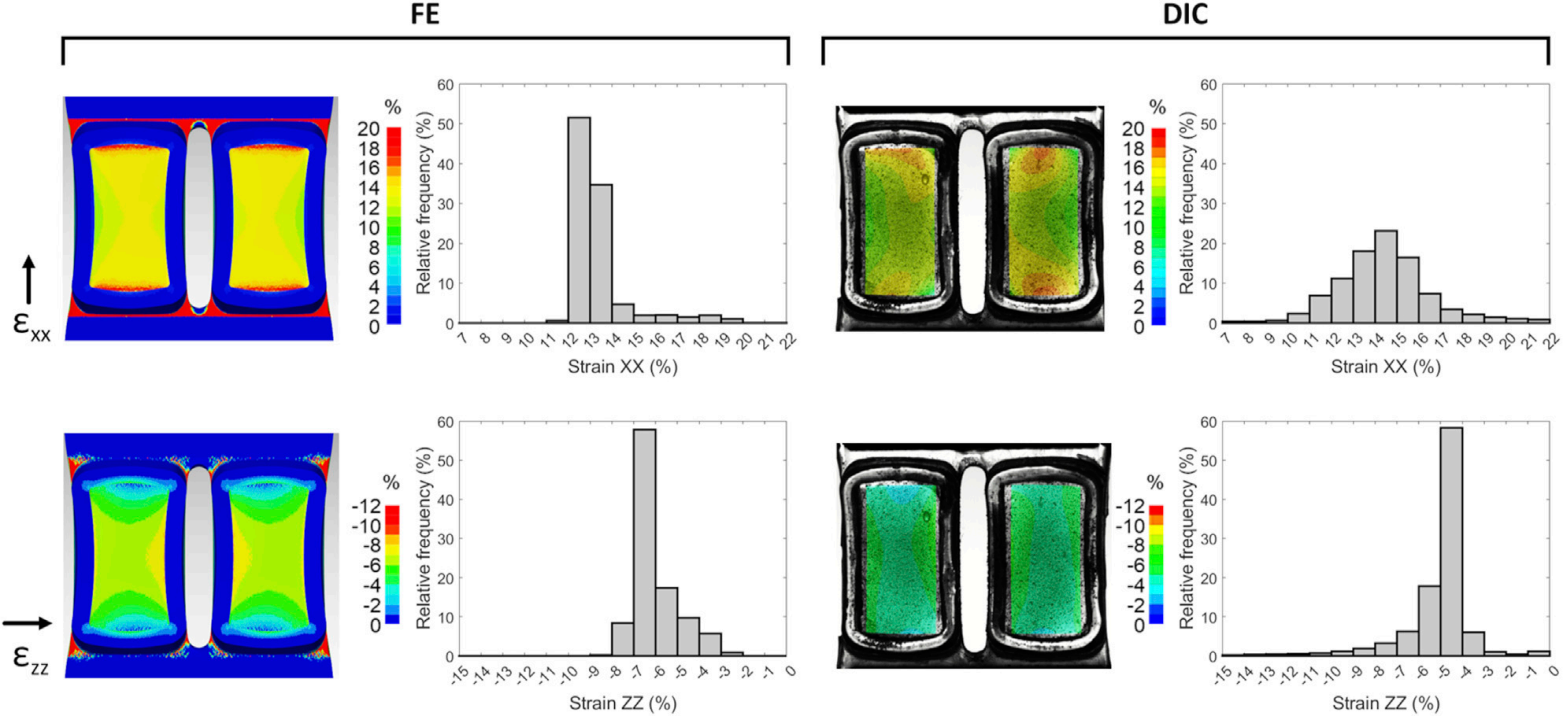
Strain distributions at the S3 well bottom at the maximum stretch obtained from FE (left) and DIC (right) analyses, for components parallel (ε_xx_) and perpendicular (ε_zz_) to the stretching direction. Histograms show the relative frequencies of the strain values, which were averaged over the stretch cycles for DIC analysis.

On the selected S3 version, refined computational FE analyses and experimental characterization tests were performed and compared. In detail, Figure 5 shows the strain distributions at the S3 well bottom obtained from FE and DIC analyses. The computational analysis yielded mean strain values of 13.3% and −5.9% for ε_xx_ and ε_zz_, respectively, showing similar strain distributions with respect to the preliminary FE analysis but with higher strain values, probably due to the different boundary conditions imposed at the clamping zones. Regarding the DIC analysis, the speckle pattern applied to the substrate surface enabled accurate strain mapping, thereby enabling reliable DIC measurements. In particular, DIC tests showed a mean strain of 14.4% ± 1.1% and −5.2% ± 0.2% for ε_xx_ and ε_zz_, respectively. The mean strain values of computational and experimental approaches are summarized in Table 1. Differences between the computational and experimental ε_xx_ values were not statistically significant (n = 3, *p* = 0.22). Observing the two wells, slight strain differences can be detected, likely due to minor irregularities in the manufactured substrate. The histograms of the strain relative frequencies revealed a slight discrepancy between FE and DIC analyses. In the stretch direction, peak values ranged between 12% and 13% for FE and 14%–15% for DIC analyses, with the latter also exhibiting a more normally distributed strain pattern (strain range approximately 11%–17% for DIC analysis and 12%–14% for FE analysis). Notably, about 83% of the actual strain values along the stretch direction fell within the range of 11%–17%, indicating that the majority of cells would be exposed to comparable levels of mechanical stimulation when using the S3 version. Looking at the strain perpendicular to the stretch direction, the DIC strain peaks presented slightly lower values compared to the FE analysis.

**TABLE 1:** Mean strain values at the maximum stretch at the S3 well bottom modelled by FE analysis and measured by DIC analysis. Experimental values are expressed as mean ± standard deviation.

Strain components	ε_xx_ (%)	ε_zz_ (%)
Imposed	15.0	n.d.*
Modelled (FE analysis)	13.3	−5.9
Measured (DIC analysis)	14.4 ± 1.1	−5.2 ± 0.2

*not defined.

Furthermore, the experimental tests revealed a stiffening of the substrate due to the sterilization process by autoclave, reaching a constant value after the second sterilization cycle (Supplementary Figure S1).

### 3.2 Biological outcomes

#### 3.2.1 Comparison among hPDLSCs and ASCs cultured under P-90s/6 h stretching protocol

**FIGURE 6:**
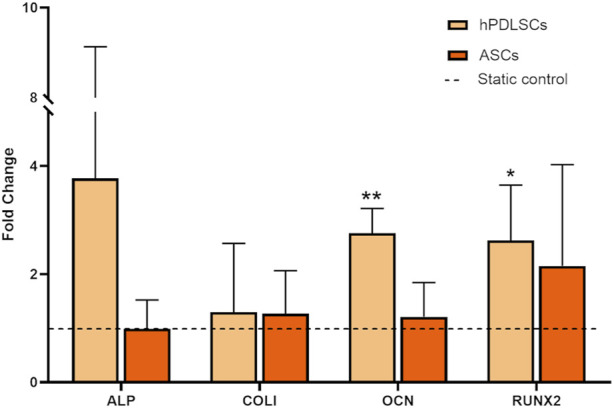
Gene expression of hPDLSCs and ASCs cultured under P-90s/6 h stretching protocol (8% of constant strain plus 7% cyclic strain (1 Hz, triangular waveform) for 90 s every 6 h for 3 days). The dashed line indicates static controls. *p < 0.05, **p < 0.01 between stimulated group vs. static control.

Preliminary biological tests were performed on hPDLSCs and ASCs to investigate the effects on different cell types of an intermittent stretching protocol (P-90 s/6 h) designed to alternate constant and dynamic stretch for mimicking the continuous intrinsic strain of the periodontium and the dynamic stretching associated with mastication (Jong et al., 2017). The tests revealed distinct responses to intermittent stretching between the two cell types. Specifically, when cultured under intermittent stretching and compared to the static control, hPDLSCs exhibited a statistically significant increase in the expression of the osteogenic genes OCN (2.71-fold increase, *p* < 0.01) and RUNX2 (2.55-fold increase, *p* < 0.05). Additionally, an increasing trend was observed in the expression of ALP (3.68-fold increase) and COL1 (1.20-fold increase), although these changes did not reach statistical significance (Figure 6). Differently, ASCs cultured under identical conditions did not exhibit any significant changes in the expression of osteogenic genes (0.99-fold change for ALP; 1.27-fold increase for COL1; 1.21-fold increase for OCN; 2.15-fold increase for RUNX2; Figure 6), thus ASCs were not exposed to further stretching protocols.

#### 3.2.2 Assessment of the early biological response of hPDLSCs cultured under three different intermittent stretching protocols

**FIGURE 7:**
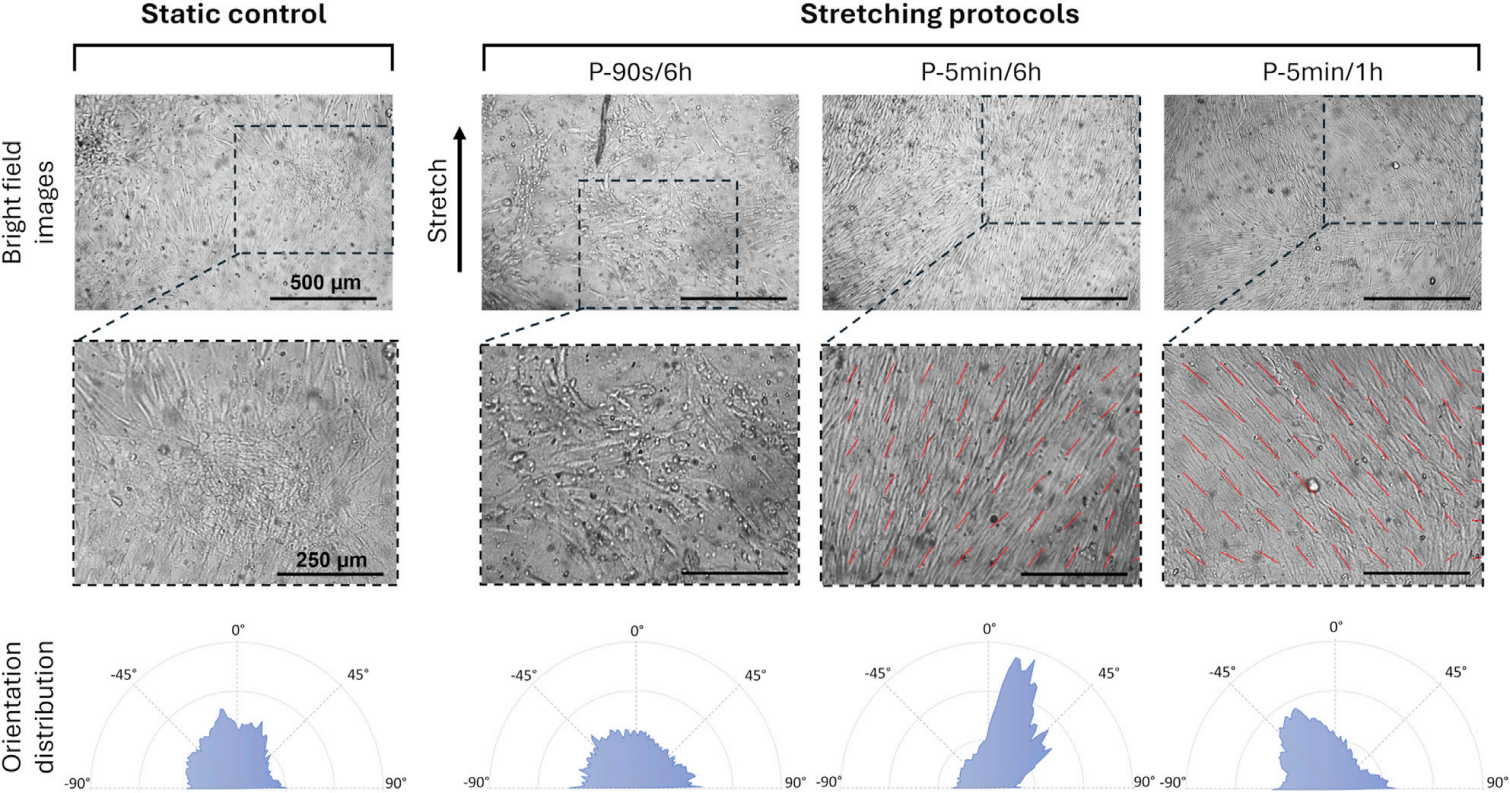
Spatial orientation of hPDLSCs exposed to different stretching protocols. Bright field images of the hPDLSCs adherent on the substrate (top, scale bar = 500 μm for all the images), with the details of cell orientation (middle, scale bar = 250 μm for all the images), and corresponding orientation distribution (bottom), depending on the applied culture condition and stretching protocol.

**FIGURE 8:**
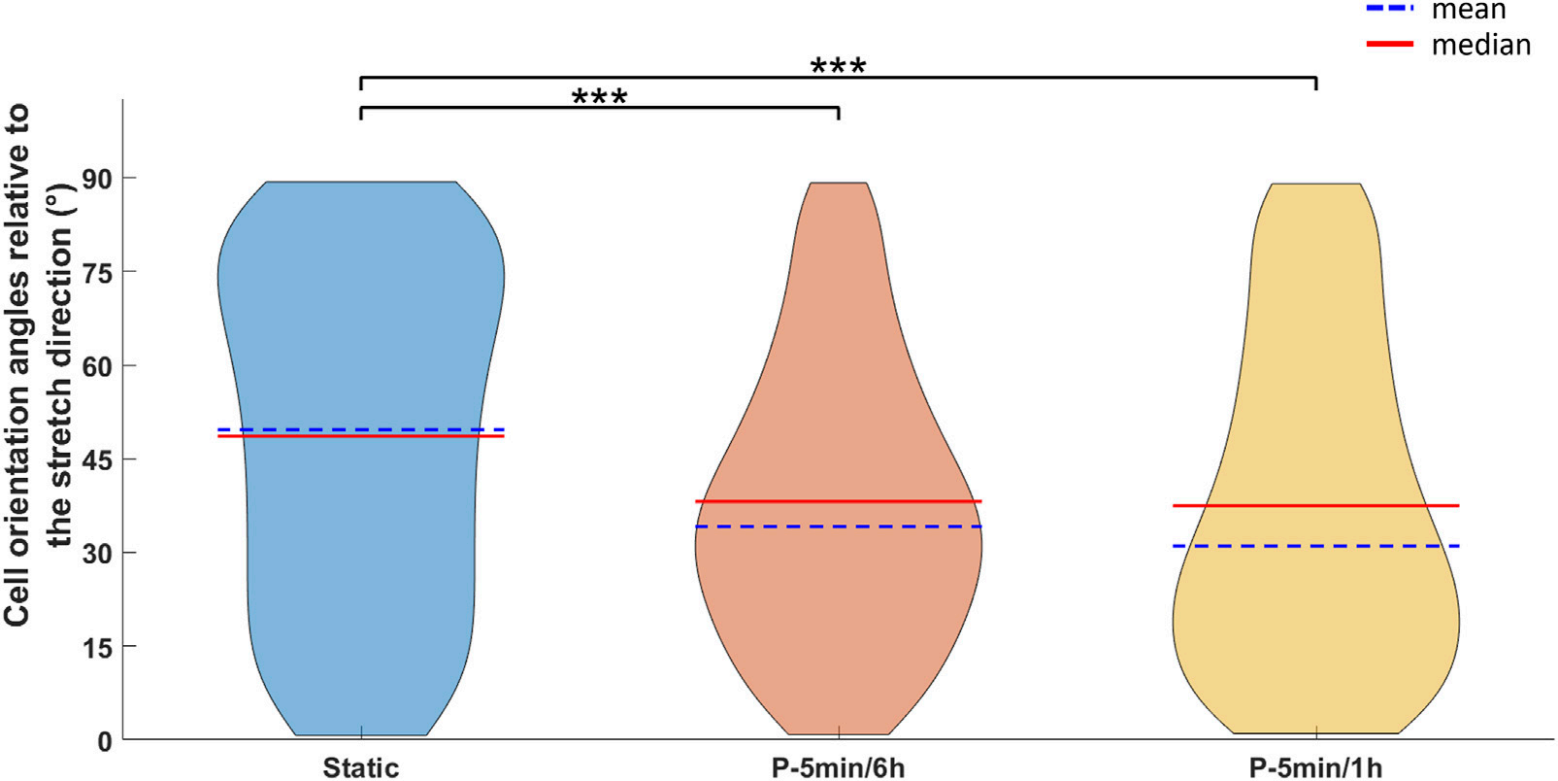
Violin plots showing the distribution of cell orientation angles (in degrees) relative to the stretch direction (corresponding to 0°) for cells cultured under static, P-5 min/6 h, and P-5 min/1 h conditions. For each distribution, the mean (blue dashed line) and median (red line) values are indicated. Statistical analysis was performed using the Kruskal-Wallis test followed by the Dunn-Šidák *post hoc* test (***p < 0.001).

To investigate the influence of the cyclic stretching associated with mastication on the early biological response of hPDLSCs, two additional intermittent stretching protocols (P-5 min/6 h and P-5 min/1 h) were tested. The analysis focused on cellular orientation and on the potential modulation of osteogenic and tenogenic gene expressions. Interestingly, the preliminary analysis of cell orientation performed on one representative image per condition revealed that, compared to the P-90 s/6 h, increasing the total amount of cyclic stretch delivered daily led to the alignment of hPDLSCs along a predominant direction (Figure 7). Indeed, particularly under the P-5 min/6 h, the computed orientation distribution revealed a peak at approximately 10°, with 0° corresponding to the stretch direction. Under the P-5 min/1 h protocol, a mild orientation of −30° was detected. Differently, hPDLSCs stimulated under P-90 s/6 h protocol presented a randomly distributed cell orientation. The statistical analysis of the cell orientation distributions, performed for the static, P-5 min/6 h, and P-5 min/1 h conditions, confirmed that intermittent stretching stimulation influences cell orientation. In particular, both stimulation protocols resulted in a statistically significant difference in cell orientation compared to the static control (*p* < 0.001), with cells preferentially oriented toward the stretch direction (Figure 8). The mean values of cell orientation angles relative to the stretch direction were 34.1° for P-5 min/6 h and 31.0° for P-5 min/1 h, compared to 49.7° for the static control. No significant difference was detected between the two stimulated groups.

**FIGURE 9:**
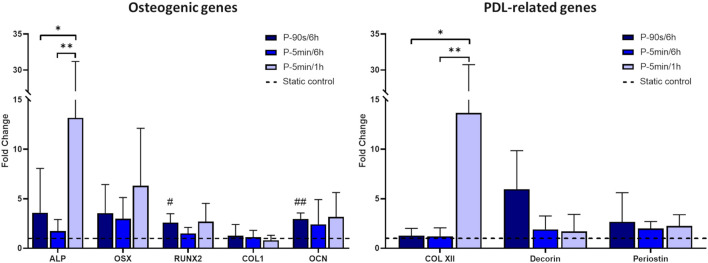
Expression of osteogenic and PDL-related genes for hPDLSCs cultured under different stretching protocols. The dashed line indicates static controls. *p < 0.05, **p < 0.01 between stimulated groups; #p < 0.05, ##p < 0.01 between hPDLSCs cultured under stretch and related static control.

The real-time PCR analysis of osteogenic and PDL-related genes (Figure 9) revealed that the P-5 min/1 h protocol, which exposed hPDLSCs to more frequent intervals of cyclic stretching, led to a significant increase in the expression of ALP and COL XII genes compared to the other stretching protocols. Moreover, although not statistically significant, all protocols resulted in an increasing trend in the expression of the osteogenic genes ALP, OSX, RUNX2, and OCN in comparison to static control. Concerning the PDL-related genes, an increasing trend in the expression of decorin and periostin was observed after stimulation with P-90 s/6 h, compared to the static control condition. On the other hand, longer exposure to the intermittent stretch stimulation did not induce any change in the expression of decorin and periostin.

## 4 Discussion

Over the past two decades, extensive evidence has demonstrated that mechanical cues arising in the cellular microenvironment can trigger a wide range of biological responses and play a pivotal role in regulating cellular behavior and tissue function *in vivo* (Bukoreshtliev et al., 2013). However, several mechanisms underlying mechanosensing and mechanochemical transduction processes remain poorly understood, mostly due to the challenges associated with applying defined loading conditions to cells and tissues cultured in traditional two-dimensional systems. In the context of developing more physiologically relevant models and effective regenerative strategies, it becomes crucial to accurately replicate the *in vivo* microenvironment, including both biochemical factors and physical stimuli. By incorporating controlled dynamic mechanical cues, *in vitro* platforms can serve as powerful technological tools for gaining deeper insights into how cells sense and respond to specific stimuli. This, in turn, will enhance the predictive power of preclinical research and support the *in vitro* development of functional tissue constructs for regenerative therapies.

In light of this, the technological aim of this study was the development of an *in vitro* research platform, based on the combination of a custom-designed deformable substrate and a stretch bioreactor, for culturing and investigating adherent cells under controlled and tunable intermittent stretch conditions. From the biological point of view, we used the platform for investigating the early biological response of hPDLSCs and ASCs to intermittent stretching protocols, with a focus on the influence of mastication-associated cyclic stretching on hPDLSCs.

Initially, three versions of the deformable substrate (S1, S2, and S3), each featuring two parallel wells, were designed and mechanically investigated adopting a computational approach. The first exploratory analysis identified the S3 version as the optimal geometry. Indeed, thanks to the presence of a gap between the wells, the S3 design guarantees symmetric strain distribution, negligible out-of-plane deflection, and uniform strain across the bottom surface of each well. Moreover, while enabling experimental parallelization, it provides a broad adhesion surface suitable for culturing enough cells for meaningful biological assessments. The S3 version was then manufactured by PDMS casting (Friedrich et al., 2017). To gain a deeper insight into the strain distributions at the S3 well bottoms and, consequently, into the mechanical stimuli generated *in vitro*, a combined computational and experimental mechanical characterization was performed (Cook et al., 2016; Subramanian et al., 2017; Zhao et al., 2022). A mean strain value of 13.3% and 14.4% ± 1.1% at the well bottom along the stretch direction (ε_xx_) was obtained from the FE and DIC analyses, respectively. The difference between the computational and experimental strain values in the stretch direction was not statistically significant (n = 3, *p* = 0.22), confirming the agreement between the computational and experimental findings, and could be ascribed to minor geometric inconsistencies in the manufactured substrate (e.g., variations in thickness). Moreover, this may also contribute to the greater variability observed in the experimentally obtained strain values (11%–17%) compared to the computational results (12%–14%), along with the influence of uncontrollable environmental factors during experimental measurements, such as variations in substrate compression intensity at the clamping system. Notably, the discrepancy between the desired (15%) and the actual strain achieved (13%–14%) highlights the importance of thoroughly characterizing the substrate deformation. Indeed, when direct monitoring of the applied physical stimulus is challenging, an accurate preliminary characterization of the platform is essential to avoid drawing misleading conclusions about the relationship between cellular responses and the stimuli provided. Concerning the strain perpendicular to the stretch direction (ε_zz_), negative values were observed both computationally and experimentally, corresponding to a substrate necking. This behavior was expected, as PDMS exhibits a positive Poisson’s ratio and therefore contracts laterally when stretched longitudinally.

As regards the bioreactor, the control software was updated for delivering fully automated intermittent stimulation protocols, featuring customizable stimulation–rest intervals without the need for user intervention. To the best of our knowledge, this platform is among the few capable of providing highly tunable mechanical stimulation thanks to its automation, which ensures high flexibility in setting intermittent stretching conditions.

The preliminary biological tests were designed to evaluate whether intermittent stretching elicits distinct responses in different cell types. The hPDLSCs were selected as a case study because, *in vivo*, they are continuously exposed to various mechanical stimuli, including stretch, making them particularly relevant for evaluating both mechanobiological responses, and their osteogenic potential in view of future applications in periodontal regeneration strategies. Conversely, ASCs were chosen as a benchmark, as they represent a non-dental cell type that has been widely investigated for regenerative medicine applications (Al-Ghadban et al., 2022). Considering the native environment of the hPDLSCs and the mechanical loading associated with mastication, a culture protocol delivering intermittent stretching stimulation was implemented. Initially, inspired by de Jong and colleagues (Jong et al., 2017), a static pre-stain of 8% was applied for replicating the intrinsic strain of the periodontium, combined with an additional intermittent 7% cyclic strain delivered for 90 s every 6 h for mimicking the dynamic stretching associated with mastication, over 3 days (P-90s/6 h). Interestingly, mesenchymal stem cells derived from different tissues, namely, the periodontal ligament and adipose tissue, responded differently to the same stimulation protocol. Regarding hPDLSCs exposed to intermittent stretch stimulation, a significant increase in the expression of the osteogenic genes OCN and RUNX2 was observed together with an increasing trend in the expression of the osteogenic markers ALP and COL1, despite the high standard deviation values likely attributable to donor-to-donor variability, according to literature (Papadopoulou et al., 2016; Oortgiesen et al., 2012; Chen et al., 2015; Roato et al., 2022). Indeed, considering that hPDLSCs are located *in vivo* in proximity of the alveolar bone, a physiological environment closely associated with bone formation and maintenance, it is plausible that they are inherently prone to undergo osteogenic differentiation. Differently, ASCs did not exhibit any significant osteogenic responses, being generally less effective at generating bone tissue than hPDLSCs, in accordance with previous studies (Oortgiesen et al., 2012; Chen et al., 2015; Citterio et al., 2020), thus ASCs were not exposed to further stretching protocols.

After assessing the osteogenic impact of the intermittent stretching on hPDLSCs, two additional intermittent stretching protocols (P-5 min/6 h and P-5 min/1 h) were tested to further investigate the influence of cyclic stretching associated with mastication on the hPDLSC early response. In detail, when the cyclic stretching stimulus was applied for a longer duration (5 min vs. 90 s) and at more frequent intervals (every 1 h vs. every 6 h) in comparison to P-90 s/6 h, a preferential cellular orientation toward the stretching direction was observed, indicating a mechanosensitive reorganization of the cells in response to the mechanical cues. In accordance with our findings, Wang and co-workers (Wang et al., 2023) reported the alignment of hPDLSCs following 12 h of stretch stimulation and Yang et al. showed that mechanical stimulation resulted in orientation of hPDLSCs along the direction of stretch (Yang et al., 2016). Focusing on the osteogenic markers, the increasing trend in their expression was also confirmed under the additional stimulation protocols, with the most pronounced effect observed following the application of cyclic stretch for 5 min every 1 h. Similarly, concerning the PDL-related markers, our study revealed a pronounced increase in COL XII expressions under P-5 min/1 h protocol, which represents the most intensive stimulation protocol tested. Interestingly, COL XII was reported to be responsive to mechanical stress and being involved in tissue repair and regeneration (Izu and Birk, 2023). These findings suggest that P-5 min/1 h protocol might promote both the osteogenic differentiation and regenerative potential of hPDLSCs, in line with observations from previous studies (Liu et al., 2020).

This study presents several limitations. As regards the actual mechanical behavior of the deformable substrate under uniaxial stretch, the strain distribution at the bottom of the optimized substrate (version S3) is not completely uniform. However, our findings indicate that approximately 83% of strain values along the stretch direction fall within the range of 11%–17%, indicating that the majority of cells are exposed to comparable levels of mechanical stimulation when using the S3 version. Therefore, while some degree of experimental variability is inevitable, the final design of the deformable substrate was considered well-suited for integration into the previously developed bioreactor and for the intended application, as it offers an effective balance between mechanical performance, bioreactor design constraints, and biological relevance, thereby supporting the suitability of the proposed research platform for *in vitro* mechanobiological studies. Moreover, although the deformation of the PDMS substrate was thoroughly characterized and enabled a direct comparison between predicted and experimentally measured mechanical conditions, the actual mechanical stimulus experienced by the cells in each individual sample was not directly measured and was instead assumed to correspond to that of the underlying substrate. Intrinsic geometric, biological, or experimental variability may indeed alter the effective transmission of mechanical cues to the cells. In this context, implementing real-time DIC analysis during culture could serve as an imaging-based sensing system, enabling the assessment of the actual strain experienced by the cells. Integrated within a closed feedback loop, this approach could further support the active regulation of mechanical loading to ensure consistent and precise stimulation levels. However, implementing such a feature involves several practical and technological challenges, including integration with cell culture equipment, optical constraints, and the need for rapid data acquisition and processing, which fall beyond the scope of the present study. Further limitation lies in the relatively low number of biological replicates, due to the inherent difficulty of obtaining primary hPDLSCs from individual donors. Nevertheless, while increasing the number of biological replicates would enhance the statistical power of our findings, the statistically significant differences observed in gene expression markers and cell orientation distributions support the robustness of the detected early cellular responses. Regarding the applied stimulation protocols, it should be acknowledged that the imposed *in vitro* intermittent stretching does not fully replicate the complexity of the *in vivo* mastication cycle, during which cells are subjected to a combination of compressive, tensile, and shear forces. Nevertheless, by applying a combination of constant and cyclic strain, our approach provided a more physiologically relevant mechanical environment compared to the purely static or purely cyclic loading conditions commonly reported in the literature. Additionally, this setup allowed for the isolation and investigation of the effects of a specific, well-controlled mechanical stimulus, namely, stretching and in particular cyclic stretching, on the cellular response. Finally, the limitations commonly associated with the use of PDMS, such as poor cell adhesion and variability in its mechanical properties, were effectively addressed in this study. Cell adhesion was improved by coating the substrate wells with type I collagen, while variability in mechanical performance was minimized through a controlled in-house fabrication procedure and by assessing the effects of the sterilization process prior to performing the mechanical characterization of the substrate.

Overall, the developed bioreactor-based platform is capable of delivering fully automated intermittent stretching protocols with adjustable stimulation–rest intervals, allowing for the alternation of static and dynamic culture conditions without the need for user intervention. Notably, for the first time, three different intermittent stretching protocols were applied to hPDLSCs and directly compared using the same experimental set-up, enabling a focused investigation into how specific dynamic culture conditions influence the biological response of the hPDLSCs. The versatility of the platform was essential to the proposed experimental design, as it enabled the investigation of cellular responses that are difficult to capture using continuous or non-automated stimulation protocols. This capability was crucial for gaining new insights into hPDLSC-specific mechanobiological responses and for advancing the development of physiologically relevant *in vitro* models for PDL tissue engineering.

## 5 Conclusion

In this study we developed a bioreactor-based platform for delivering automated intermittent stretch stimulation to adherent cells *in vitro*, integrating a stretch bioreactor with a custom-designed PDMS deformable substrate. Through a combination of computational and experimental analyses, we showed that the actual strain experienced by the substrate, and assumed to be experienced by the adherent cells, can differ from the imposed strain, highlighting the importance of accurately characterizing the actual applied mechanical stimulus to correlate it with the cellular biological response. Moreover, we used the platform for investigating the early biological response of different stem cells, i.e., hPDLSCs and ASCs, to intermittent stretching protocols. We firstly revealed that intermittent stretching can induce distinct early modulation of gene expression, with hPDLSCs exhibiting upregulation of osteogenic gene expression. Furthermore, we demonstrated that the timing of the cyclic stretching can play a crucial role in promoting cellular differentiation and orientation and enhancing the regeneration capability of hPDLSCs.

Overall, our findings confirm the versatility of the proposed platform as a valuable tool for investigating the effect of controlled stretching on mechanosensitive cells. In addition, the study provides meaningful insights into the mechanobiological interplay between applied intermittent stretching and hPDLSCs responses, emphasizing their potential for applications in PDL tissue engineering.

## Data Availability

The datasets presented in this study can be found in online repositories. The names of the repository/repositories and accession number(s) can be found below: https://doi.org/10.5281/zenodo.15497986, 15497985.
