# Supplementary material for: A bioreactor-based platform for investigating the early response of human periodontal ligament stem cells to intermittent mechanical stretching
Source: Front Bioeng Biotechnol. 2025 Sep 3;13:1634143. doi: 10.3389/fbioe.2025.1634143 (PMC12441168; doi:10.3389/fbioe.2025.1634143)
Supplement: Supplementary file 1 [file DataSheet1.pdf]

A bioreactor-based platform for investigating the early response of human periodontal ligament stem cells to intermittent mechanical stretching

Giovanni Putame[1,2†], Beatrice Masante[1,2,3†], Marta Tosini[1,2], Andrea T. Lugas[1,2], Ilaria Roato[3], Mara Terzini[1,2], Alberto L. Audenino[1,2], Federico Mussano[3Δ], Diana Massai[1,2Δ*]

[1]Department of Mechanical and Aerospace Engineering and Polito[BIO]Med Lab, Politecnico di Torino, Turin, Italy

[2]Interuniversity Center for the Promotion of the 3Rs Principles in Teaching and Research, Turin, Italy

[3]Bone and Dental Bioengineering Lab, Department of Surgical Sciences, CIR-Dental School, University of Turin, Turin, Italy

† These authors contributed equally to this work and share first authorship.
[Δ] These authors share last authorship.

*** Correspondence:**
Diana Massai
diana.massai@polito.it

Supplementary Material

Supplementary Table 1. Parameters of the digital image correlation (DIC) analysis adopted for the experimental characterization of the substrates.

DIC Software	Vic-2D, v. 6.2.0, isi-sys GmbH
Camera-Target distance	690 mm
Field of view	50 mm x 30 mm
Lens aperture	f/5.6
Frame rate	25 frames/s
Correlation subset size	73 - 113 pixels
Correlation step size	24 - 30 pixels
Correlation strain filter size	15 data points
Pixel size	0.012 mm

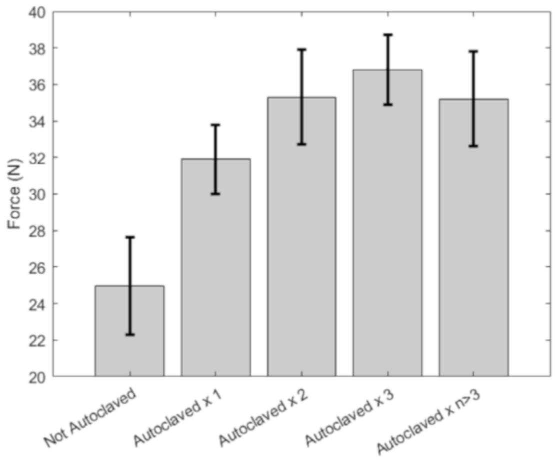

Supplementary Figure 1. Variation in the maximum traction force needed to stretch the substrate by 3 mm after subsequent autoclave sterilization cycles (20 min at 121 °C). It can be noted a stiffening of the substrate due to autoclave sterilization. The mechanical behaviour of the substrate becomes stable after 2 sterilization cycles.
